# Rethinking gene regulatory networks in light of alternative splicing, intrinsically disordered protein domains, and post-translational modifications

**DOI:** 10.3389/fcell.2015.00008

**Published:** 2015-02-26

**Authors:** Karl J. Niklas, Sarah E. Bondos, A. Keith Dunker, Stuart A. Newman

**Affiliations:** ^1^Plant Biology Section, School of Integrative Plant Science, Cornell UniversityIthaca, NY, USA; ^2^Department of Molecular and Cellular Medicine, Texas A&M Health Science CenterCollege Station, TX, USA; ^3^Center for Computational Biology and Bioinformatics, School of Medicine, Indiana UniversityIndianapolis, IN, USA; ^4^Department of Cell Biology and Anatomy, New York Medical CollegeValhalla, NY, USA

**Keywords:** cell fate specification, combinatorial transcription regulation, eukaryotes, evolution, development, gene regulatory networks, multi-stable dynamical systems, protein structure

## Abstract

Models for genetic regulation and cell fate specification characteristically assume that gene regulatory networks (GRNs) are essentially deterministic and exhibit multiple stable states specifying alternative, but pre-figured cell fates. Mounting evidence shows, however, that most eukaryotic precursor RNAs undergo alternative splicing (AS) and that the majority of transcription factors contain intrinsically disordered protein (IDP) domains whose functionalities are context dependent as well as subject to post-translational modification (PTM). Consequently, many transcription factors do not have fixed cis-acting regulatory targets, and developmental determination by GRNs alone is untenable. Modeling these phenomena requires a multi-scale approach to explain how GRNs operationally interact with the intra- and intercellular environments. Evidence shows that AS, IDP, and PTM complicate gene expression and act synergistically to facilitate and promote time- and cell-specific protein modifications involved in cell signaling and cell fate specification and thereby disrupt a strict deterministic GRN-phenotype mapping. The combined effects of AS, IDP, and PTM give proteomes physiological plasticity, adaptive responsiveness, and developmental versatility without inefficiently expanding genome size. They also help us understand how protein functionalities can undergo major evolutionary changes by buffering mutational consequences.

“The unpredictable and the predetermined unfold together to make everything the way it is. It's how nature creates itself, on every scale, the snowflake and the snowstorm.”

–Tom Stoppard, *Arcadia*, Act 1, Scene 4

## Introduction

A fundamental assumption of contemporary developmental biology is that gene regulatory networks (GRNs, herein defined as circuits of interacting transcription factors and their cis-acting regulatory elements) are primary mechanisms controlling development. According to this assumption, at any time, the relative levels of transcription factors in an extended network determine the progress of development by regulating downstream genes (Carroll et al., 2004; Davidson and Erwin, 2006). This conception of gene control in multicellular organisms, which was formulated in several related versions a half century ago (Britten and Davidson, 1969; Kauffman, 1969; Britten, 1982; Davidson, 1982), proposes that GRNs are deterministic dynamical systems exhibiting multiple stable states. The theoretical foundations of this framework can be traced to studies of the bi-stable gene regulatory switch between the lytic and lysogenic states of the lambda phage in *Escherichia coli* (see Ptashne, 2004), and have been generalized and applied to the larger and more elaborate GRNs of eukaryotes in the form of models ranging from discrete Boolean networks to continuous systems of ordinary differential equations (Glass and Kauffman, 1973; Kauffman, 1974; Savageau, 1974; Glass, 1975; Lauffenburger, 2000; Jaeger and Monk, 2014). A commonality among these models describing cell differentiation is the assumption that gene products (i.e., proteins, particularly transcription factors) have specific identities and connectivity relationships to one another in the GRNs in which they function (Hasty and Collins, 2001; Forgacs and Newman, 2005). According to this view, variation in the outcome of the function of GRNs (e.g., alternative cell types) arises from nonlinearities and stochastic effects to which such complex, deterministic systems are subject.

This paradigm has been extended to other gene expression mechanisms that have been characterized since the GRN dynamics model was first proposed. Among these mechanisms is the alternative splicing (AS) of pre-mRNA exons and introns to assemble different proteins, a process that permits variation in the functionalities of subsets defining components of GRNs (e.g., transcription factors) at the level of RNA processing. Although the mixing and matching of basic system components has no direct counterpart in a truly deterministic GRN model, the factors controlling AS can be viewed as having well defined functionalities, since the associated GRN dynamics permit different cell fates in a combinatorial deterministically prescribed manner. Likewise, the modulatory effects of microRNAs, riboswitches, and the enzymes that mediate post-translational modifications (and that can silence genes) can be viewed as adding a complicating, yet still deterministic set of regulatory mechanisms.

Here, we propose that this perspective must also contend with evidence that the majority of eukaryotic transcription factors contain intrinsically disordered protein (IDP) domains (Liu et al., 2006; Dunker et al., 2014) that comprise almost two-thirds of their sequences (Ward et al., 2004; Minezaki et al., 2006a,b; Singh and Dash, 2007; Xie et al., 2007), and with the fact that the conformations of these domains, and hence their functions, are contingent on the intra-and extracellular environments in which these proteins function (Ducas and Rhoades, 2014; Srinivasan et al., 2014). Consequently, the specificity of the binding of most regulatory transcription factors to cis-regulatory elements, as well as their partnering with other factors mediating conditional responses to cellular physiological status, are context dependent and subject to change even in the absence of genetic or epigenetic alterations. Importantly, the functions of IDPs are modulated further by both alternative splicing (AS) and post-translational modifications (PTMs), especially phosphorylation (Iakoucheva et al., 2004; Romero et al., 2006; Singh and Dash, 2007). For example, AS, IDPs, and PTMs are known to act synergistically in modulating the activities of the tumor repressing transcription factor p53 (Dunker et al., 2008) and to underlie the functions of several other proteins crucial for the evolution of multicellular organisms (Dunker et al., 2014; Niklas et al., 2014). The combined functional consequences of AS, IDPs, and PTMs make modeling GRN dynamics as strictly deterministic systems incomplete at best (Kupiec, 2009; Braunschweig et al., 2013). If transcription factors do not have fixed cis-acting regulatory element targets, but rather can alter their specific identity and network-topological status within a given GRN depending on other proteins in the nucleus and external environmental factors, it follows that GRNs can no longer be viewed as deterministic systems in a strict physical or mathematical sense. If our conceptualization is correct, we predict that the incorporation of AS, IDP, and PTM (designated, collectively but as operatively independent processes, as AS–IDP–PTM) and their well-documented synergistic interactions into an expanded (and thus more computationally sophisticated) approach will provide deeper insight into recently recognized genotype-phenotype mapping anomalies, e.g., developmental system drift (True and Haag, 2001) and the puzzle of missing heritability (Zaitlen and Kraft, 2012).

Toward this goal, we present evidence that AS–IDP–PTM promotes alternative, context-dependent GRN states, and thus serves a critical role in a broad range of cellular responses, including cell fate specification. We also present evidence that these three components are ancient in eukaryotic GRNs, a speculation driven by the observation that early divergent unicellular eukaryotes achieve temporally alternative physiological and reproductive states and respond adaptively to contingent environmental conditions by virtue of AS–IDP–PTM. Further, we provide suggestions for how the determinate outcomes of plant and animal development are realized despite the indeterminacy of isolated GRNs. Our conclusion is that we face an “incompleteness theorem” when we attempt to reduce development to a single causal level (Niklas and Kutschera, 2012).

Finally, it is important to emphasize that rather than proposing AS-IDP-PTM as a developmental mechanism in its own right, we see its collective role as creating an adaptive plasticity that significantly diminishes the strict determinism that some attribute to GRNs. The latter framework is all-too-often treated as a biological *Welterklärung* (theory of everything) simply because the usual experimental frames of reference make it difficult to think beyond genes and their interactions. We hope to establish that the synergistic interactions among AS, IDP, and PTM have enabled living systems to evolve beyond the constraints that are inevitable in regulatory networks that depend on single-level dynamics.

## Alternative splicing (AS)

Alternative splicing produces protein isoforms from the same precursor mRNA by retaining or excluding different exons to achieve differential translation. First observed in the infectious adenovirus cycle (Berget et al., 1977; Chow et al., 1977) and subsequently in the transcripts of normal, endogenous genes (Leff and Rosenfeld, 1986), AS occurs in all eukaryotic lineages (Black, 2003) and becomes more prevalent as complexity, estimated by the number of different cell types, increases (Chen et al., 2014a). Although a number of scenarios have been advanced for the origin of AS, including a role in enabling the cell to filter out aberrant transcripts (Catania and Lynch, 2008), we suggest that the connection between cell type number and AS (given the association with IDP and PTM) is an inherent one that promoted occupation of new niches (see below “AS-IDP-PTM Phylogenetic Patterns”).

**Figure 1:**
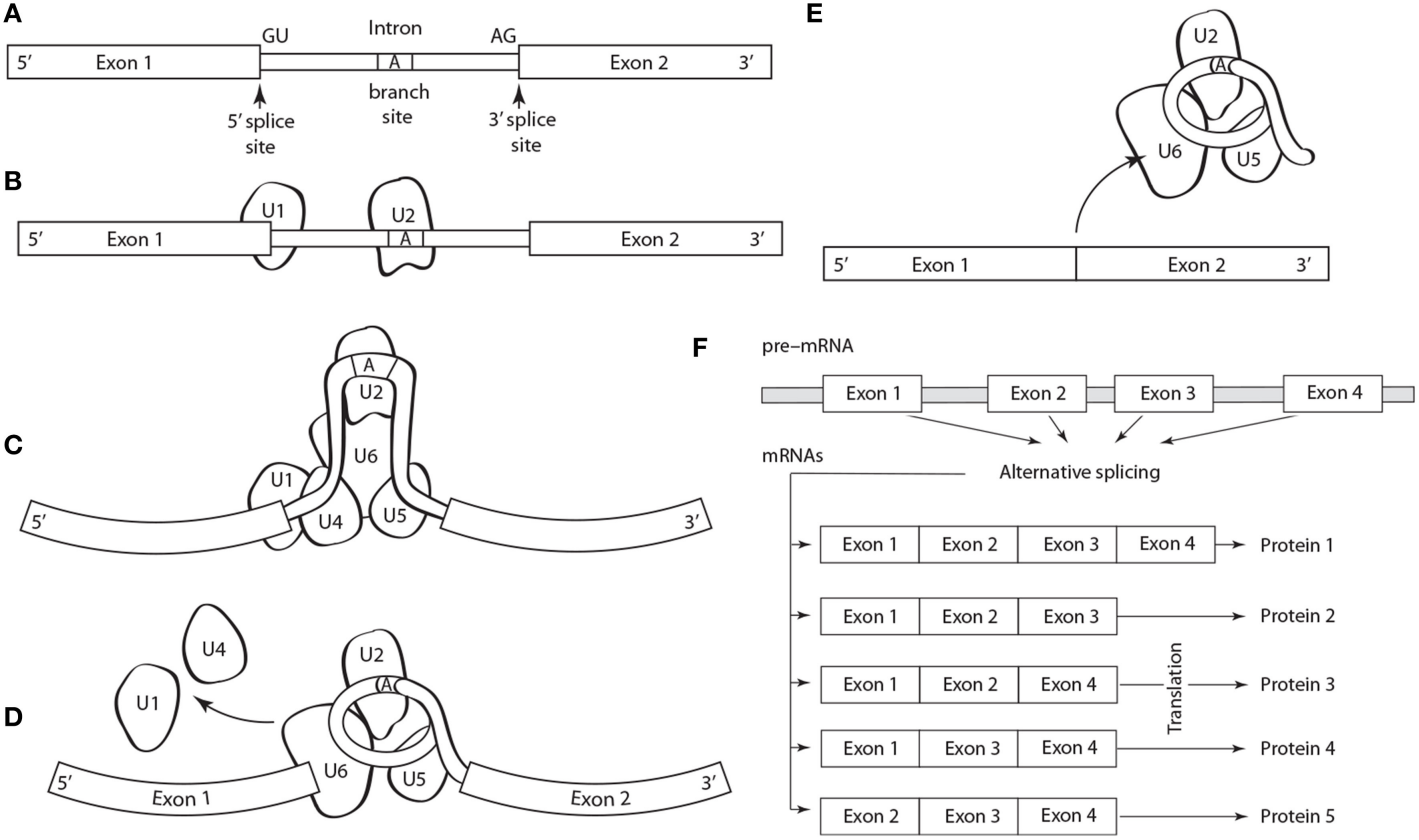
**Schematic of the structure and operation of a spliceosome to remove an intron flanked by exons on a pre-mRNA**. **(A)** The splicing process is guided by a highly conserved 5′ splice site GU sequence, an A branch site near a pyrimidine-rich region, and a 3′ splice site AG sequence. The spliceosome protein complex contains RNA and protein components (i.e., small nuclear ribonucleoprotein or snRNPs, designated U1, U2, U4–U6) that recognize and bind to the pre-mRNA conserved sequences in a stepwise process. **(B)** The process begins with U1, which binds to the 5′ splice site, and U2, which binds to the **(A)** branch site. **(C)** U4, U6, and U5 subsequently bind the pre-mRNA transcript forming the mature spliceosome complex that configures the intron into loop bringing the 5′ and 3′ splice sites converge. **(D)** The mature spliceosome splices the 5′ first and the 5′ GU end second, creates a lariat by connecting the 5′ end to the A branch site. The U1 and U4 snRNPs are released and the 3′ splice site is cleaved. **(E)** The intron, U3 and the U5–U6 ensemble are released, and exons are attached. The intron will degrade and the snRNPs will be reused. **(F)** Schematic of alternative splicing of a pre-mRNA with four exons that can yield five different proteins.

Five basic types of alternative splicing exist: alternative 3′ acceptor site, alternative 5′ donor splice site, intron retention, mutually exclusive exon splicing, and exon skipping (Black, 2003). The last is the most frequent. Regulation and selection of the splice sites are performed by trans-acting splicing activator and repressor proteins within an RNA–protein complex, the spliceosome, which is canonically composed of five small nuclear RNAs (i.e., U1, U2, U4–U6) and a range of assorted protein factors (Figure 1). Splicing is regulated by trans-acting repressor-activator proteins and their corresponding cis-acting regulatory silencers and enhancers on the pre-mRNA (Matera and Wang, 2014). The effects of splicing factors are often position-dependent (Barash et al., 2010). A splicing factor that functions as an activator when bound to an intronic enhancer element may function as a repressor when bound to its splicing element in the context of an exon (Lim et al., 2011).

The secondary structure of the pre-mRNA transcript also determines which exons and introns will be spliced, e.g., by bringing together splicing elements or by masking a sequence that would otherwise serve as a binding element for a splicing factor. Consequently, activators, repressors, and secondary pre-mRNA structure constitute a splicing “code” that defines the protein isoforms produced under different cellular conditions. Additionally, the elements within this code function interdependently in ways that are context dependent, both intracellularly and extracellularly (Talavera et al., 2013). For example, cis-acting regulatory silencers and enhancers are influenced by the presence and relative position of other RNA sequence features, and the trans-acting context is affected by intracellular conditions that are in turn influenced by external conditions (Chen et al., 1999; Wang et al., 2004; Matlin et al., 2005) and other RNA sequence features. Furthermore, some cis-acting elements may reverse the effects on splicing if specific proteins are expressed in the cell (Boutz et al., 2007; Spellman et al., 2007). Indeed, the number of factors influencing AS is significantly large. A recent, empirically successful computer model for predicting the number and type of spliceoforms in different human tissues depends on nearly 1400 exonic and intronic features and identifies more than 20,000 unique single-nucleotide variants that likely affect splicing (Xiong et al., 2015).

AS is adaptive and highly conserved. There is strong selection against mutations that alter splicing (Fairbrother et al., 2004; Ke et al., 2008). For example, Chang et al. (2014) report a conserved AS pattern for heat shock transcription factors in the moss *Physcomitrella patens* and the flowering plant *Arabidopsis thaliana* and show that the AS mechanism for heat regulation among land plants is an ancestral condition. Using mRNA sequence data, Pan et al. (2008) report that transcripts from ≈95% of human multi-exon genes undergo alternative splicing and that ≈100,000 intermediate to high abundance AS events occur in different tissue systems. Similar results are reported by Johnson et al. (2003) using microarray analyses of human tissues.

In addition to producing protein isoforms, AS produces a disproportionate number of transcription factors with intrinsically disordered protein (IDP) domains, which leads to a synergistic expansion of functional and regulatory diversity (Liu et al., 2006; Vuzman and Levy, 2012). In the case of the *Drosophila* protein Ultrabithorax (Ubx), different spliceoforms have different affinities to common target sequences. Consequently, the isoforms are not interchangeable during development (Reed et al., 2010). More generally, when GRN-related transcription factors are alternatively spliced the function of the GRN may vary in a spatiotemporal fashion under the influence of physiological and physical factors external to the network.

## Intrinsically disordered protein (IDP) domains

Intrinsically disordered protein (IDP) domains often form small interaction surfaces characterized by multiple specificity and modest affinity, an enhanced binding diversity, the ability to form large interaction surfaces with high affinity, fast association and dissociation rates, polymorphism in the bound state, and wide range of intracellular lifetimes (Dunker and Uversky, 2010; Oldfield and Dunker, 2014). These traits make IDPs versatile signaling and regulatory molecules. Studies have identified intrinsically disordered domains as enriched in the non-constitutive exons, indicating that protein isoforms may display functional diversity due to the alteration of tissue-specific modules within these regions (Buljan et al., 2012). IDP domains can exist as molten globules with defined secondary structure or as unfolded chains that can function through transitions among different folded states. Their functional conformations can change by binding to other proteins and nucleic acids (Uversky, 2002; Oldfield et al., 2008; Hsu et al., 2013). IDPs also contribute to the process of alternative splicing: the RS-repeat domains of the conserved SR family of metazoan splicing factors are intrinsically disordered (Braunschweig et al., 2013). Post-translational modifications can also alter IDP functionalities (Iakoucheva et al., 2004; Dyson and Wright, 2005; Oldfield et al., 2008).

Numerous examples of IDP domains involved in transcriptional regulation are known (Campbell et al., 2000; Haynes and Iakoucheva, 2006; Liu et al., 2006; Sun et al., 2013). The C-terminal activation domain of the bZIP proto-oncoprotein c-Fos, which effectively suppresses transcription *in vitro*, is intrinsically disordered and highly mobile (Campbell et al., 2000). The C-terminal domain of the transcriptional co-repressor CtBP, which facilitates gene targeting and coordinated histone modifications in the multi-protein complex, is intrinsically disordered (Bhalla et al., 2006; Haynes and Iakoucheva, 2006; Sun et al., 2010). The unbound N-terminal domains of the DELLA proteins, which are central to the integration of plant developmental and environmental signaling, undergo disorder-order transitions upon binding to interacting proteins (Sun et al., 2010). The DELLA proteins are similar in their domain structures to the GRAS protein family, whose N-domains are intrinsically disordered (Sun et al., 2011) and are extensively involved in plant signaling by virtue of undergoing disorder-order transformations in interactions with a variety of molecular partners involved in development, light signaling, nodulation, and auxin signaling and transcription regulation to biotic and abiotic stresses.

Metazoans also carry out intercellular signaling via small molecules, called nuclear hormone receptors (NHRs), that bind to their cognate proteins. Following ligand binding, NHRs translocate to the nucleus where they act as transcription factors. In addition to the structured ligand and DNA binding domains, these NHRs have flanking and linking IDP domains that bind to large numbers of partners. These domains may be responsible for the variable or context dependent responses following hormone signaling (Simons and Kumar, 2013). Thus, NHRs use disorder to bind to many partners, and many partners use disorder to bind to structured docking sites on NHR ligand binding domains (Mohan et al., 2006; Dunker et al., 2014).

Another important example is provided by the Wnt pathway. This key signaling pathway, which is utilized in development from sponges to flies to mammals (Cadigan and Nusse, 1997; Nusse and Varmus, 2012), employs both IDPs and PTMs in fundamental ways. Briefly, β-catenin, a dual-function cofactor for adhesion and transcription, is phosphorylated at four nearby sites in a disordered tail by the destruction complex. This complex is held together by the disordered scaffold protein axin, which uses a long disordered region to flexibly tether β-catenin to two kinases, GSK3β and CK1α, thus speeding up the phosphorylation reactions by colocalization (Xue et al., 2012b; Dunker et al., 2014). These multiple phosphorylation events regulate both nuclear localization and proteasomal digestion of β-catenin. The activity of adenomatous polyposis coli (APC), a massively disordered member of the β-catenin destruction complex, is also regulated by phosphorylation (Minde et al., 2013). Thus, β-catenin accumulates, translocates to the nucleus, and turns on several genes that activate cell proliferation and polarity.

These examples illustrate that intrinsically disordered transcription factor domains are central to plant and animal development and homeostasis. They are by no means exceptional. Liu et al. (2006) found that 82.6–93.1% of the transcription factors in three databases contain extended regions of intrinsic disorder, in contrast to 18.6–54.5% of the proteins in two control datasets. Focusing on human transcription factors and using a disorder predictor and Hidden Markov Models to search for regions that are homologous to structured protein domains, Minezaki et al. (2006b) report that only 31% of the transcription factor residues align with known structured domains, which is only half of the 62% structurally aligned residues for *E. coli* proteins that regulate transcription.

Since protein-DNA recognition and protein-protein recognition are central transcription factor functionalities, these and other studies illustrate the extent to which eukaryotic transcription factors manifest extensive flexibility as a consequence of disorder-associated signaling and transcriptional regulation (Dunker et al., 2014). This permits them to bind to a greater array of partners that in turn can induce conformational changes in bound protein and DNA substrates (Oldfield et al., 2005). A well-studied example of this is the isoforms of the *Drosophila* Ubx transcription factor described above. Here, two intrinsically disordered domains modulate the binding affinity of the structured DNA-binding homeodomain to its target sequence (Liu et al., 2008) and to other transcription factors (Johnson et al., 1995; Bondos et al., 2006; Hsiao et al., 2014). The C-terminal IDP region, which is alternatively spliced, alters the relative affinity of Ubx for different DNA sequences (Liu et al., 2009).

## Post-translational modifications (PTMs)

Post-translational modifications (PTMs) alter the regulatory interfaces of proteins so as to induce gain, loss, or exchange of binding partners, thereby affecting function at many levels (Van Roey et al., 2013). Significantly, the structure of chromatin, the mechanochemical medium within which eukaryotic transcription occurs, is regulated by PTM of histone proteins. Mediator, a multi-protein complex involved in RNA Pol II-regulated transcription, is both positively and negatively regulated by phosphorylation (Gonzalez et al., 2014). Combinatorial PTMs of the C-terminal domain of RNA Polymerase II regulate multiple stages of transcription initiation and coordinate transcription with mRNA processing (Yogesha et al., 2014).

However, our focus here is on the effect of PTMs on specific transcription factors. In transcriptional regulation, each transcription factor must participate in many different macromolecular recognition/binding events (Bondos and Tan, 2001; Sun et al., 2013; Abdel-Hafiz and Horwitz, 2014). Transcription factor binding to DNA often occurs in conjunction with other specific transcription factors, requiring tissue-specific protein-protein interactions as well. Transcription factors must interact with Mediator or other components of the general transcription apparatus to elicit their function. Many transcription factors that are active in developmental processes also bind histone acetylases and de-acetylases. Phosphorylation can regulate each of these events. For example, DNA binding by the transcription factor Ets-1 is allosterically coupled to a serine-rich region (Lee et al., 2008; Mooney et al., 2014). Ca^2+^ signaling induces phosphorylation of this region, which modulates DNA binding by Ets-1. Phosphorylation of the intrinsically disordered PAGE4 protein (as part of the stress-response pathway) causes PAGE4 to release the transcription factor c-Jun, enabling its activity in transcription regulation (Mooney et al., 2014). Phosphorylation can also increase interactions among cofactors. For example, the cytokines TNF and IL-1 induce phosphorylation of the p65 subunit of NF-κ B, which in turn induces a conformational change that allows p65 ubiquitination and interaction with transcriptional cofactors (Milanovic et al., 2014). Association of Elk-1 and ETS domain transcription factors with Mediator and histone acetyltransferases is dependent on Elk-1 phosphorylation (Galbraith et al., 2013).

As a final example, we again turn to the *Drosophila* Hox protein Ubx (Ronshaugen et al., 2002). This transcription factor is multiply phosphorylated (Gavis and Hogness, 1991), including at sites within its intrinsically disordered domain, which regulates DNA binding, protein-protein interaction and transcription activation (Tan et al., 2002; Bondos et al., 2006; Liu et al., 2008, 2009). Given that phosphorylation has the potential to regulate as well as coordinate multiple transcription factor functions, it is not surprising that this mechanism is widely used. Indeed, transcription factors are disproportionately phosphorylated compared to other classes of proteins (Kaganovich and Snyder, 2012). Furthermore, the divergence of the sequence and function of transcription factor paralogs created by whole genome duplication events correlates positively with the extent to which the transcription factor is phosphorylated (Kaganovich and Snyder, 2012).

## Synergistic effects of AS-IDP-PTM

Importantly, although AS, IDP, and PTM can operate independently of one other, they are more often co-localized to operate synergistically. The co-localization of AS, IDP, and PTM is apparent in many ways. For example, pre-mRNA segments undergoing AS are far more likely to code for IDP domains than for structured domains. These AS-associated IDP domains also frequently contain binding sites for protein or nucleic acid partners such that they operate together to “rewire” GRNs (Romero et al., 2006; Dunker et al., 2014). The AS–IDP collaboration to rewire GRNs is commonly observed at the tissue-specific level and is well conserved over evolutionary time (Buljan et al., 2012, 2013; Ellis et al., 2012; Colak et al., 2013).

IDP domains are also far more likely than structured regions to undergo PTMs, especially the phosphorylation of serines and threonines (Iakoucheva et al., 2004; Gao et al., 2010; Gao and Xu, 2012). These IDP-associated PTMs are often observed to alter partner choice for IDP-based protein-protein interactions (Oldfield et al., 2008; Hsu et al., 2013), which can further rewire GRNs. In addition, different patterns of multiple PTMs in localized protein regions have been shown to signal different downstream results, leading to their designation as a histone or PTM “code” (Strahl and Allis, 2000; Lothrop et al., 2013). Finally, “constellations” of multiple PTMs generally occur in IDP regions, (Pejaver et al., 2014), some examples of which have been shown to be further modified by AS (Dunker et al., 2008).

## As–IDP–PTM phylogenetic patterns

Evidence drawn from phylogenetically different lineages indicates that AS–IDP–PTM is ancient and has undergone significant amplifications during the prokaryotic-to-eukaryotic transition. For example, analyses of the Viridiplantae (the green and charophycean algae, and the land plants) show that early divergent unicellular chlorophytes employ AS extensively and that the frequency of AS in the unicellular green alga *Chlamydomonas reinhardtii* is comparable to that of the flowering plant *A. thaliana* (Labadorf et al., 2010). Many of the ancient physiological processes in the Viridiplantae rely on IDPs, e.g., the extensively disordered N-terminal region of the CP12 protein regulates two critical (and extremely ancient) enzymes in the Calvin cycle (glyceraldehyde-3-phosphate dehydrogenase and phosphoribulokinase) (Mileo et al., 2013). More generally, in a comprehensive study using over 39 million expressed sequence tags available for 47 eukaryotic species with fully sequenced genomes, Chen et al. (2014b) found that the occurrence of AS has increased steadily over the last 1.4 billion years of eukaryotic evolution. The frequency of AS is not due to covariance with other factors proposed to account for organismic complexity, e.g., genome size, protein interactivity, and proteome disorder. These authors conclude that organismic complexity, as gauged by the number of different cell types, has increased as a result of AS driven transcript diversification that has increased the information content of cells (Chen et al., 2014b).

Less is known about the extent to which IDP–PTM has changed over evolutionary history. Quantitative measures of proteome intrinsic disorder are only recently becoming available. However, a positive relationship between a large number of proteins with intrinsically disordered domains and the extent to which species are evolutionarily derived has been noted. This relationship appears to be step-wise rather than continuous, which likely reflects major evolutionary transitions. Xue et al. (2012a) examined 3484 viral, bacterial, and eukaryotic proteomes and found that the largest variance of intrinsically disordered content occurred among the viruses (i.e., 7.3–77.3%), whereas only a weak correlation between complexity as gauged by the number of different cell types and overall ID domain content was observed within the eukaryotes. These authors also report that the ID domain content is generally independent of proteome size for both the prokaryotes and eukaryotes, but that it is significantly higher for eukaryotic compared to prokaryotic species and possibly correlated with the more elaborate signaling systems eukaryotes use to coordinate their intracellular functions (Xue et al., 2012a). Schad et al. (2011) report that complexity (as gauged by the number of cell types) and proteome size (measured as the total number of amino acids) correlate positively across diverse organisms, and that the fraction of ID domains increases significantly from prokaryotes to eukaryotes, but does not increase further within the eukaryotes.

However, in contrast to the aforementioned study, which did not delve into a species-level analysis of the data, Niklas et al. (2014) have uncovered a statistically robust (*r*^2^ = 0.721, *P* < 0.0001, *F* = 44.0) log-log linear relationship between the number of different cell types and the fraction of ID residues in the proteomes reported for a diverse group of unicellular and multicellular algae, land plants, invertebrates, and vertebrates (spanning genera such as *Chlamydomonas, Volvox, Arabidopsis, Hydra, Caenorhabditis, Drosophila, and Homo sapiens*). Perhaps more significant, the slope of this log-log linear relationship numerically significantly exceeds unity, which indicates that a small increase in the fraction of proteomic ID residues is correlated with disproportionately large increases in the diversity of cell types. As in the Schad et al. (2011) study, Niklas et al. (2014) found that the slope for the log-log linear relationship between the number of different cell types and genome size (as gauged by base-pair numbers) is less than unity, which is consistent with the so-called C paradox. Clearly, as noted by many workers, statistically robust correlations between any two variables of interest are not evidence for cause-effect relationships. Nevertheless, strong correlations can be taken as evidence for consistency between empirical observations and theoretical expectations.

## Resolving stochastic developmental effects

**Figure 2:**
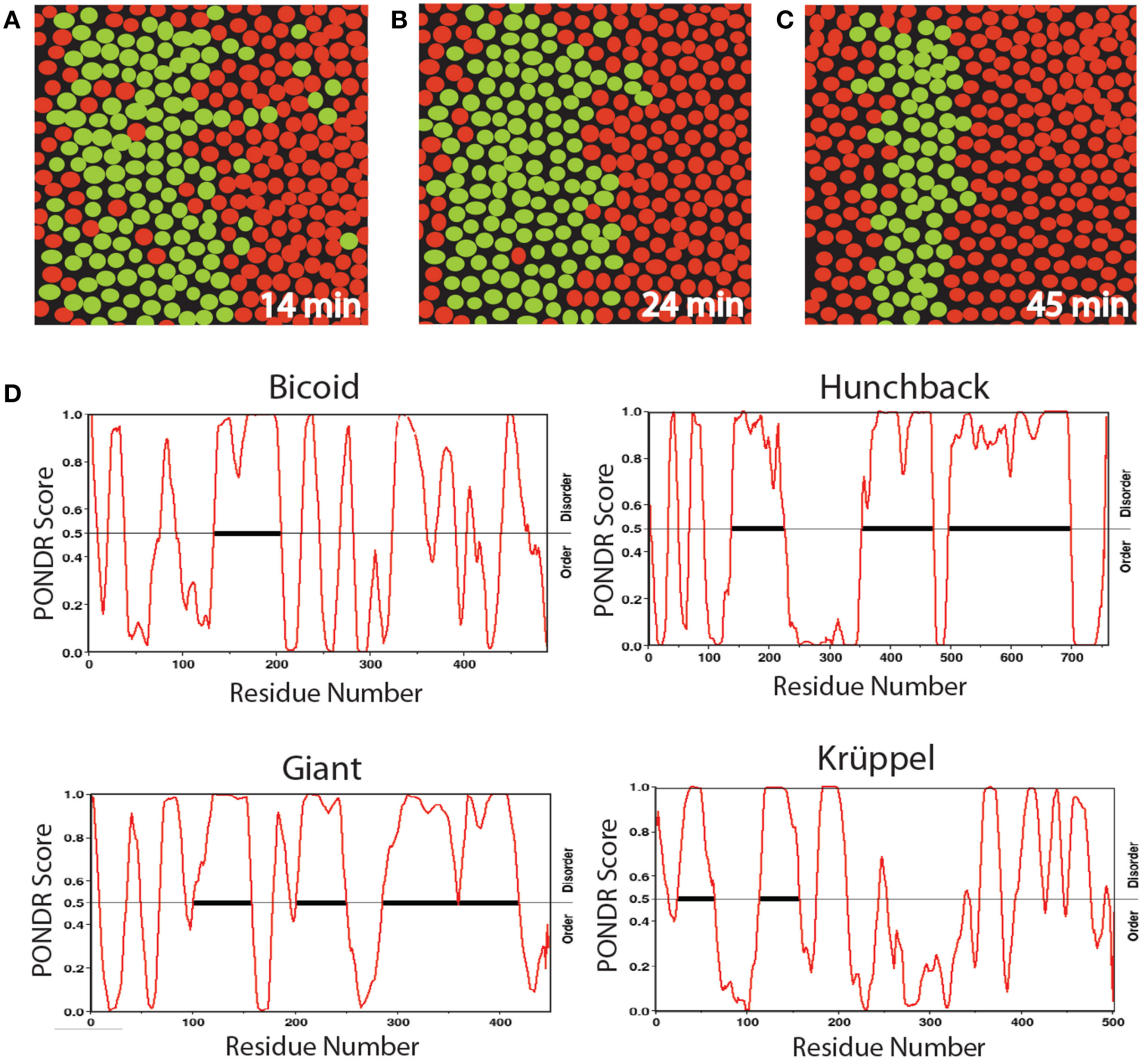
**Diagrams of a section of *Drosophila* embryo expressing the eve>MS2 reporter at three different times in nc14 centered at ≈37% embryo length (A–C) and PONDR scores for order vs. disorder in four transcription factors (D)**. Nuclei that show foci of active transcription are depicted in green. **(A–C)** Redrawn from Bothma et al. (2014, see their Figures 2A–B).

Many developmental processes appear initially disorganized but subsequently produce an ordered, patterned structure. Live images of *Drosophila* embryos provide striking examples of this phenomenology (Bothma et al., 2014). For example, fluorescent imaging has been used to monitor the genesis of the second stripe of *eve* expression in *Drosophila*, a critical step in the segmentation stage of development (Figures 2A–C). *Eve* is initially activated in a broad stripe, in which cells expressing *eve* are mixed with cells lacking *eve* expression. This observation reflects an initial randomness of the initial decision to transcribe (or not) the *eve* gene. However, over time, the eve expression domain is refined to a narrow stripe of homogeneous eve-expressing cells. The initial dynamic behavior is due in part to short bursts of *eve* transcription characterized by a range of Pol II loading rates, indicative of non-deterministic behavior. Although deterministic systems often exhibit transient behavior en route to achieving their stable states, the *eve* patterning mechanism is not conventionally deterministic. Whereas the maternally deposited transcription factor Bicoid is essential for the correct spatial positioning of eve stripe 2, and the latter's target CREs in the stripe 2 *eve* enhancer are well-characterized (Ludwig et al., 2011), studies have shown that disrupted, unstable, and highly abnormal Bicoid gradients fail to disturb the precision of this process (Lucchetta et al., 2008).

We propose that the initial heterogeneity in the *eve* patterning system is related to the effects of AS-IDP-PTM on the transcription factors regulating this gene, which is activated by Hunchback and repressed by Giant and Krüppel. According to a recent computational analysis (Ilsley et al., 2013), Bicoid has a dual context-dependent activator/inhibitor role in eve 2 expression, although the mechanism for this is unknown. All four transcription factors contain large intrinsically disordered domains (Figure 2D). In addition, all four transcription factors are phosphorylated and bicoid is alternatively spliced (Ollo and Maniatis, 1987; Capovilla et al., 1992). The variation in transcription levels during these burst phases could be the result of regulation by different spliceoforms or phosphoforms of these proteins.

These issues have been explored in greater detail for Ubx, where the nature of intrinsically disordered domains provides a basis for both this early stochastic behavior, and mechanisms to resolve such behavior into an ordered response. The alternative splicing and phosphorylation of Ubx are tissue-specific, creating different dominant forms of the transcription factor in each tissue with tissue-specific capacities for protein interactions, DNA binding, and transcription regulation (Gavis and Hogness, 1991; Liu et al., 2008; Kim et al., 2010; Reed et al., 2010; Fuxreiter et al., 2011; de Navas et al., 2011). However, in any given cell minor forms created by splicing and phosphorylation are also present, yielding a mixture of Ubx functional states (O'Connor et al., 1988; Gavis and Hogness, 1991; Lopez and Hogness, 1991). In our model, the form of the Ubx protein that first binds a newly available gene target is expected to determine the initial response, creating an initial variation in transcription levels and stochastic phenotypes.

**Figure 3:**
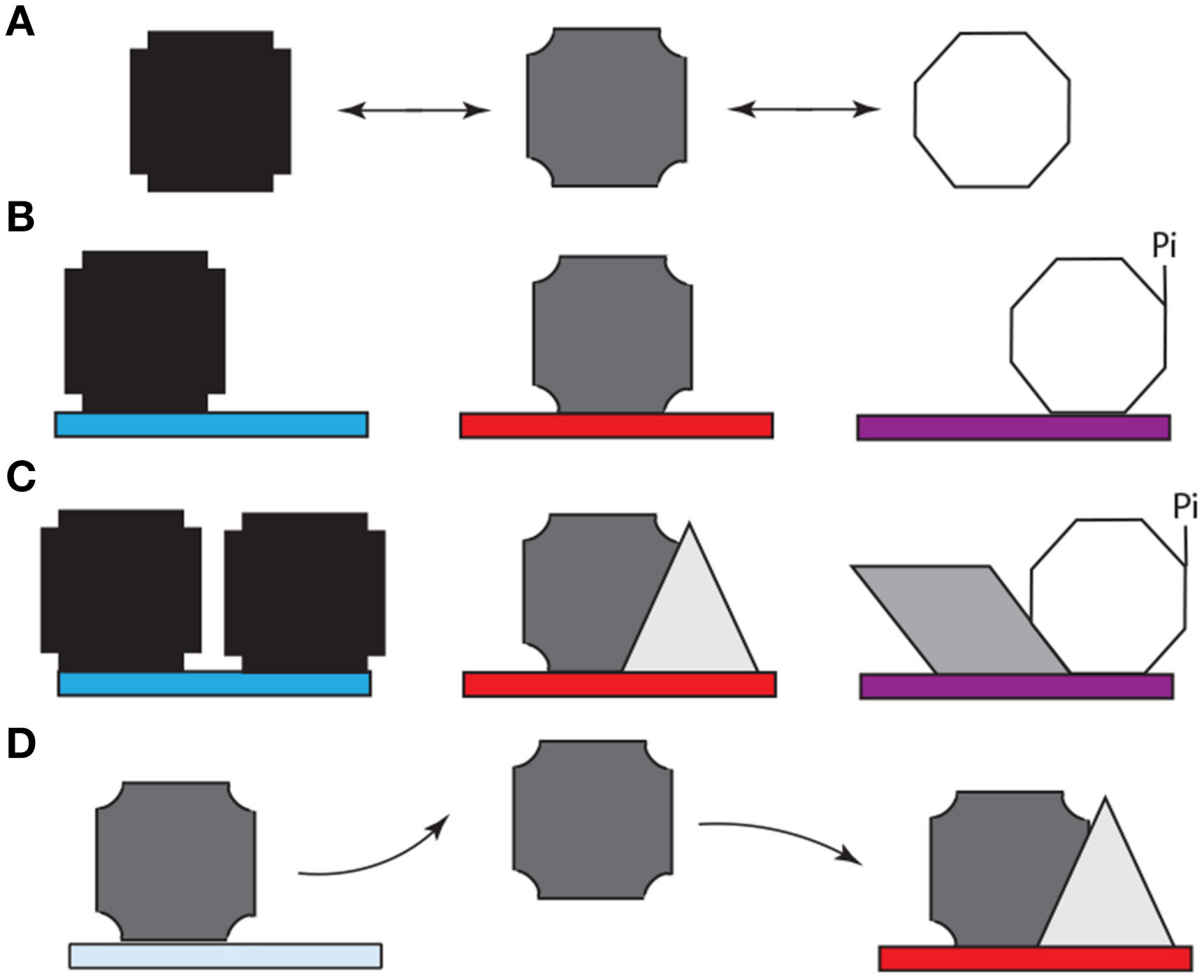
**Model for the role of alternative splicing, intrinsically disordered protein domains, and post-translational modification (e.g., phosphorylation) in cell-specific DNA target site selection by Hox proteins. (A)** The structured DNA binding homeodomain of Hox proteins binds to a variety of DNA sequences with extremely high affinity (Liu et al., 2009). Most Hox protein sequences are intrinsically disordered and thus can adopt a variety of conformations (represented by different polygons), which rapidly interconvert. **(B)** Specific spliceoforms and phosphoforms of a Hox protein are produced in each tissue. The variants can reinforce a subset of Hox conformations or enable access to new conformations. Specific conformations may enhance or inhibit affinity for particular DNA sequences (denoted by different colored rectangles). **(C)** When a Hox protein binds a “correct” DNA sequence, additional copies of the same Hox proteins, or additional other transcription factors (represented by different new polygons). These proteins bind both the Hox protein and neighboring DNA binding sites, thus reinforcing Hox-DNA binding. Alternately, the Hox protein isoform can bind other proteins first, followed by DNA binding by the protein complex. **(D)** When a Hox protein binds a DNA sequence that is not appropriate for this Hox variant, an incorrect transcriptional readout is transiently produced. Both the lower intrinsic binding affinity for this site and the lack of reinforcing interactions with other transcription factors eventually cause the Hox protein to dissociate. The released Hox protein then has an opportunity to bind to a high affinity site to produce the appropriate response for this tissue.

As in the case of Bicoid (Ilsley et al., 2013), Ubx has context-dependent dual activator/inhibitor roles and its “collaboration” with other transcription factors, which is regulated in a spatiotemporal fashion, can determine the “sign” (positive or negative) of its regulatory role (Walsh and Carroll, 2007) and thus profoundly influence GRN logic. Such modulation, canalization, and refinement of the Ubx response is likely to depend on post-translational modification or protein interactions mediated by the intrinsically disordered regions of this protein. Like most transcription factors in development, Ubx (i) regulates genes encoding cell signaling proteins (Pearson et al., 2005; Bondos et al., 2006), (ii) is regulated (phosphorylated) by cell signaling proteins (Gavis and Hogness, 1991; Taghli-Lamallem et al., 2008), and (iii) binds cell signaling proteins and cell signaling-regulated transcription factors (Liu et al., 2008). These mechanisms enable the community of cells to make a collective decision regarding gene regulation. Binding by the form of Ubx that is supposed to regulate a specific target gene enhancer will be supported by the presence of other factors that cooperatively regulate this gene in conjunction with Ubx. Downstream cell-signaling events could further reinforce this decision within a neighboring group of cells. In contrast, binding by the incorrect form of Ubx may lack the requisite co-factors and signaling to stabilize the bound complex, ultimately resulting in dissociation of the protein and providing a second opportunity for the correct Ubx form to bind. In this paradigm, AS–IDP–PTM–protein interactions (i) generate the initial stochastic behavior, (ii) are required to reinforce the correct cell decisions, and (iii) mediate the rectifying response (Figure 3).

The described behaviors appear to differ from the transients exhibited by deterministic dynamical systems (such as GRNs in the standard model), as they evolve toward their “attractors,” i.e., stationary points and orbits, or in the case of Turing-type reaction-diffusion systems (reviewed in Forgacs and Newman, 2005), stationary non-uniform spatial patterns. The temporal evolution, and the distribution and stability of attractors, are strongly dependent on the network topology of such systems. In contrast, the rewiring of Bicoid and Ubx regulatory circuits en route to the biologically functional patterns in which they function suggests that, rather than determining the end-states of the respective systems, the GRNs are actually subordinate to them.

## Disorder from order

**Figure 4:**
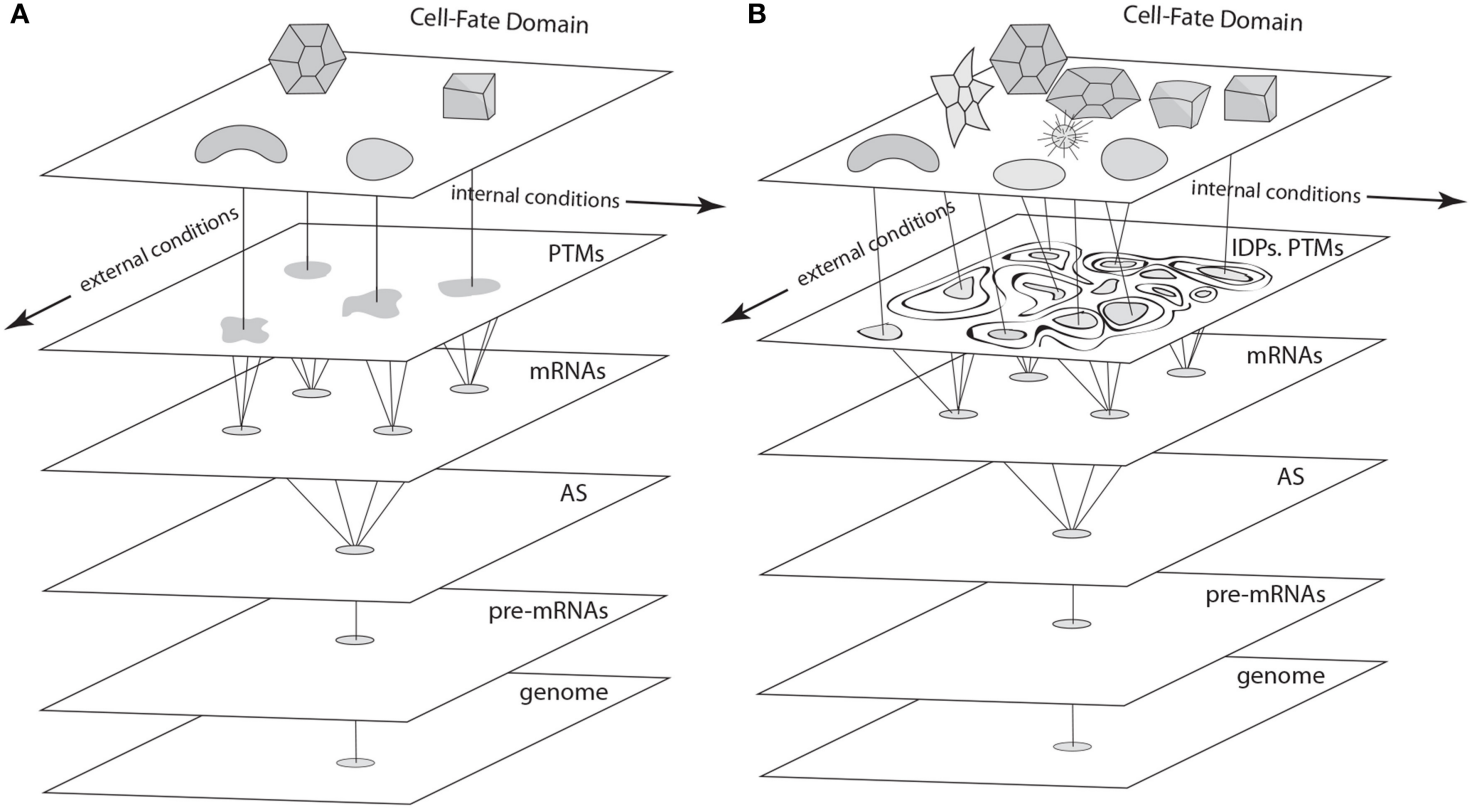
**Schematics of cell fate specification viewed from the standard deterministic GRN perspective (A) and the non-deterministic GRN perspective described in the text (B). (A)** In the standard view, pre-mRNAs undergo alternative splicing (AS), and transcription factors specified by the variant mRNAs undergo post-translational modifications (PTMs) to form a cadre of proteins involved in cell-fate specification networks (GRNs, represented as irregular shapes) via their cis-acting targets. Discrete cell types result from the deterministic properties of these GRNs. **(B)** In the proposed non-deterministic view, transcription factors are generated by AS and PTM operating in the context of intrinsically disordered protein (IDP) domains. Cell-fate determination in this case (represented by interactions among components of variable, context-dependent identity and specificity), is a consequence of the time- and spatial-context dependency of each of the levels shown in this schematic, which depend on internal and external cellular conditions in a fashion that eludes deterministic description at the level of GRNs.

According to the most mathematically sophisticated deterministic GRN dynamics models (e.g., Foster et al., 2009; Jaeger and Monk, 2014), each cell type is an attractor. That is, if a cell's state at a given time is represented by a point in a multidimensional “state space” whose axes are the concentration ranges of key transcription factors, the point's position will change until it settles stably at one of a finite number of discrete sub-regions within the space (Figure 4). These sub-regions (i.e., system attractors) can be stationary points, periodic orbits, or a mixture of these behaviors, depending on the subset of the components involved in the system. Deterministic systems of sufficient complexity can also exhibit the so-called “butterfly effect,” in which an infinitesimal displacement of the system point can take it along widely divergent trajectories, as well as chaotic behaviors, characterized by “strange attractors,” i.e., regions within the state space in which a point remains bounded but wanders in an unpredictable fashion (Strogatz, 2001; Kaneko, 2006). Each attractor in a deterministic dynamical system is surrounded by a “basin of attraction” toward which a system point gravitates. Importantly, the number of attractors within a deterministic dynamical system is, in principle, a predictable function of its network topology and rate constants, and is always much smaller than the number of basic interacting components. The rationale for applying this mathematical formalism to GRNs and cell differentiation thus arises from observations like the fact that the human genome specifies more than 1300 transcription factors (Vaquerizas et al., 2009) but the human body contains only about 244 cell types (Niklas et al., 2014).

If a system is less than fully deterministic, the dynamics become much more complicated. One example is “noisy systems” in which conventional network topologies and interactions are in place, but the values of the variables (such as concentrations of transcription factors) are perturbed by extrinsic or intrinsic factors, as a consequence of some key proteins and other biomolecules being present in small numbers in individual cells and thus varying in a stochastic fashion (Elowitz et al., 2002; Bhalla, 2004; Gomez et al., 2014). Indeed, mathematics has shown that the notion of an “attractor” still applies, but that their properties and thus behavior are less predictable than in systems without noise (Jacobs and Schreiber, 2006; Zhao and Li, 2011).

The issue of noise is well recognized to confound the biological effects of even those gene regulatory systems that are formally deterministic. For example, Rosenfeld and coworkers examined the bacteriophage lambda promoter P_R_ in *E. coli* and found that protein production rates fluctuated over the time scale of one cell cycle, with intrinsic noise levels of the circuit decaying rapidly within each cycle (Rosenfeld et al., 2005). Nonetheless, the aggregate effect of fluctuations in other cellular components undermined accuracy in transcriptional responses for time-scales longer than a single cell cycle. Thus, although individual circuits can be demonstrated to behave according to a deterministic GRN dynamics model over short time intervals (Rosenfeld et al., 2007), GRN–level determinism breaks down when embedded in the wider network of real complexity and temporal scale.

It is our contention that the gene regulatory indeterminism produced by AS-IDP-PTM is different in kind from that of deterministic dynamical systems operating (as described above) under nonlinear, chaotic, or stochastic regimes. GRNs acted upon by AS-IDP-PTM are *inherently non-deterministic*, since network logic (i.e., connectivity relationships), strengths of interactions, and even the identities of the transcription factors as regulatory molecules are constantly subject to change due to internal fluctuations and external influences. As a consequence, the properties of the transcription factor components of GRNs that have been thought to make them suitable to be represented as nodes in discrete networks or variables in systems of ordinary differential equations are actually subject to change from point to point and moment to moment during development. Although GRNs have been modeled as Boolean networks acting “at the border between order and chaos” (Shmulevich et al., 2005), the presence of AS-IDP-PTM in actual GRNs raises questions about whether any strictly deterministic models can adequately capture the behavior of these systems (Figure 4).

## Order out of disorder

As noted earlier, GRNs are frequently not deterministic due to the independent and interdependent actions of AS–IDP–PTM. Nevertheless, this is in no way equivalent to the counterfactual assertion that embryonic development is itself non-deterministic. Rather, it is our hypothesis that deterministic GRN dynamics are not a sufficient causal basis for developmental regularities. Although a GRN might provide a rough template for a cellular function (particularly if the GRN was established concurrently with the evolutionary origination of that function), remodeling of the GRN by AS–IDP–PTM will have rendered cell phenotype identity increasingly dependent on internal (i.e., cell physiological) and external (e.g., microenvironmental and extraorganismal) conditionalities beyond the GRN itself. This assertion is consistent with, if not confirmed by, somatic stem cell production and subsequent differentiation as well as examples of dedifferentiation (e.g., Sprecher and Desplan, 2008).

The conservation of a useful cell function or morphological phenotype over the course of evolution accompanied by an unmooring from its originating GRN appears to be a common scenario in the history of multicellular plants and animals, reflected in what has been termed “developmental system drift” (True and Haag, 2001; Haag, 2014). The inability to consistently pin heritable variation in diseases and other traits to particular genes (Zaitlen and Kraft, 2012), may also plausibly be a manifestation of the operation of AS-IDP-PTM over evolutionary time.

AS-IDP-PTM may also provide flexibility and adaptability even within a single tissue type. For example, in a study of lineage commitment among the eight progenitor populations of the major myeloid and lymphoid elements of human blood, Chen et al. (2014b) identified cell type-specific expression changes during early differentiation stages encompassing 6711 genes and 10,724 transcripts. They also detected 7881 novel splice junctions and 2301 differentially used AS events, enriched in genes involved in regulatory processes. Although only AS was considered, the authors concluded that “a previously undetected layer of regulation affecting cell fating... involves transcriptional isoforms switching without noticeable changes at the gene level” (Chen et al., 2014b).

Finally, AS–IDP–PTM and its synergies provide a context for understanding how the functionalities of ancient proteins and regulatory networks can be stably modified over the course of evolution to adapt to changing external conditions. Target sequence recognition and selectivity by a transcription factor are subtle properties of the latter's structure (De Masi et al., 2011). It is well documented, for example, that novel relationships between protein structure and PTM educed by mutation can lead to altered protein-protein interactions resulting in dramatic changes in transcription factor function (Brayer et al., 2011; Lynch et al., 2011). However, synergy with AS and IDP provides an even greater multiplicity of functional states that can be explored ecologically and physiologically ahead of any mutational change.

Furthermore, nascent potentially adaptive mutations can be retained within (and subsequently integrated into) GRNs by virtue of AS–IDP–PTM modifications that can buffer GRNs from the immediate consequences of such mutations. In this scenario, a mutated GRN could survive by virtue of AS–IDP–PTM adaptive modifications that would permit the GRN time to adaptively reorganize. In this way, evolutionary changes would involve an interactive “genome ⇔ AS–IDP–PTM” feedback loop. Consider the transcription factor AkUbx, a homolog of Ubx in the velvet worm *Acanthokara kaputensis*, an invertebrate with a simple body plan. AkUbx has very little intrinsic disorder and is not alternatively spliced (Grenier and Carroll, 2000; Galant and Carroll, 2002). Ubx, in contrast, which participates in the development of the later-evolving, more complex body plans of *Drosophila melanogaster* has considerable disorder content as well as undergoing AS and PTM (Gavis and Hogness, 1991; Liu et al., 2008; Reed et al., 2010). The synergistic effects of AS-IDP-PTM ensured that once it had arisen in the earliest multicellular GRNs it would have promoted its own elaboration, as well as generation of new developmental contexts that would eventually be reflected in greater anatomical and physiological complexity.

## Conclusions

The association of alternative splicing (AS) with intrinsically disordered protein (IDP) domains and post-transcriptional modifications (PTMs) is a core functional complex that mediates the modifications of protein functionalities required for context dependent cell signaling, regulation, and differentiation. The combined effects of AS-IDP-PTM also likely buffer genomes from mutations (some of which can subsequently become adaptive to new conditions) and contributes to the evolvability of GRNs (see for example, Masel and Trotter, 2010; Steiner, 2012; Albergante et al., 2014). AS–IDP–PTM is ancient and likely promoted variability and thus adaptive evolution to support more complex intracellular signaling processes coordinating the activities of functionally interdependent discretized organelles, cells, tissues, and organs.

Unlike promoter activity, which primarily regulates the amount of transcripts, AS changes the structure of transcripts and their encoded proteins. The ability of IDP domains to assume different conformations expands the functional repertoire of proteins assembled by AS from a pre-mRNA to diversify the phenotypic domain that a single genome can provide. This repertoire is yet again increased by PTMs, which generate additional functionalities. Thus, AS–IDP–PTM can yield virtually limitless combinatorial possibilities, which can be adaptively sifted over the course of evolution.

Consequently, GRNs are inherently plastic and therefore adaptive. Moreover, they function in a noisy cellular milieu owing to the operation of AS–IDP–PTM in a multitude of other biochemical pathways as well as the effects of mutations and variations in gene and protein copy number (Richard and Yvert, 2014). (Note that this noisiness is over and above the described intrinsic indeterminacy.) The evolution of cell differentiation may indeed have depended on such stochastic effects (Kupiec, 2009). However, heterogeneity at both the molecular and cell phenotypic levels must be suppressed for reliable development to occur. This is accomplished by a variety of “scaffolding” effects (Caporael et al., 2014) at multiple scales, including consistency of external cues from neighboring cells and the physical environment (Braendle and Félix, 2008), and the stabilizing effects of natural selection (Richard and Yvert, 2014).

The multiscale nature of developmental processes is increasingly acknowledged (see, for example, Schnell et al., 2008). In particular, tissue morphogenesis and cellular pattern formation involves the mobilization, by key gene products of the developmental “toolkit,” of mechanical, electrical and other physical phenomena external to the genome (Forgacs and Newman, 2005; Newman and Bhat, 2009; Hernández- Hernández et al., 2012). It is therefore unsurprising that the determination of cell type identity does not reside at the single scale occupied by GRNs, but rather draws on factors at several causal levels, as described above, among the most important of which are the mechanical aspects of chromatin reorganization associated with changes in gene expression (Amendola and van Steensel, 2014; Lavelle, 2014).

We do not suggest that deterministic mathematical and computational modeling of GRNs has nothing to contribute to understanding cell fate determination. However, this perspective must acknowledge and integrate the ubiquitous effects of AS–IDP–PTM. Just as genes *per se* have long been rejected as the exclusive or privileged level of determination of phenotype and evolutionary change, new understanding of the complexities of gene expression and the conditional identities of its protein products call into question a deterministic GRN-based reductionism in developmental and evolutionary biology.

### Conflict of interest statement

The authors declare that the research was conducted in the absence of any commercial or financial relationships that could be construed as a potential conflict of interest.

## References

[B1] Abdel-Hafiz H. A. Horwitz K. B. (2014). Post-translational modifications of the progesterone receptors. J. Steroid Biochem. Mol. Biol. 140, 80–89. 10.1016/j.jsbmb.2013.12.008 24333793 PMC3923415

[B2] Albergante L. Blow J. J. Newman T. L. (2014). Buffered Qualitative Stability explains the robustness and evolvability of transcriptional networks. eLife 3:e02863. 10.7554/eLife.02863 25182846 PMC4151086

[B3] Amendola M. van Steensel B. (2014). Mechanisms and dynamics of nuclear lamina-genome interactions. Curr. Opin. Cell Biol. 28, 61–68. 10.1016/j.ceb.2014.03.003 24694724

[B4] Barash Y. Calarco J. A. Gao W. Pan Q. Wang X. Shai O.. (2010). Deciphering the splicing code. Nature 465, 53–59. 10.1038/nature09000 20445623

[B5] Berget S. M. Moore C. Sharp P. A. (1977). Spliced segments at the 5′ terminus of adenovirus 2 late mRNA. Proc. Natl. Acad. Sci. U.S.A. 74, 3171–3175. 10.1073/pnas.74.8.3171 269380 PMC431482

[B6] Bhalla J. Storchan G. B. MacCarthy C. M. Uversky V. N. Tcherkasskaya O. (2006). Local flexibility in molecular function paradigm. Mol. Cell. Proteomics 5, 1212–1223. 10.1074/mcp.M500315-MCP200 16571897

[B7] Bhalla U. S. (2004). Signaling in small subcellular volumes. I. Stochastic and diffusion effects on individual pathways. Biophys. J. 87, 733–744. 10.1529/biophysj.104.040469 15298882 PMC1304483

[B8] Black D. L. (2003). Mechanisms of alternative pre-messenger RNA splicing. Annu. Rev. Biochem. 72, 291–336. 10.1146/annurev.biochem.72.121801.161720 12626338

[B9] Bondos S. E. Tan X. X. (2001). Combinatorial transcription regulation: The interaction of transcription factors and cell signaling molecules with homeodomain proteins in *Drosophila* development. Crit. Rev. Eukaryot. Gene Expr. 11, 145–171. 10.1615/CritRevEukarGeneExpr.v11.i1-3.80 11693959

[B10] Bondos S. E. Tan X.-X. Matthews K. S. (2006). Physical and genetic interactions link Hox function with diverse transcription factors and cell signaling proteins. Mol. Cell. Proteomics 5, 824–834. 10.1074/mcp.M500256-MCP200 16455680

[B11] Bothma J. P. Garcia H. G. Esposito E. Schlissel G. Gregor T. Levine M. (2014). Dynamic regulation of eve stripe 2 expression reveals transcriptional bursts in living *Drosophila* embryos. Proc. Natl. Acad. Sci. U.S.A. 111, 10598–10603. 10.1073/pnas.1410022111 24994903 PMC4115566

[B12] Boutz P. L. Stoilov P. Li Q. Lin C.-H. Chawla G. Ostrow K.. (2007). A post- transcriptional regulatory switch in polypyrimidine tract-binding proteins reprograms alternative splicing in developing neurons. Genes Dev. 21, 1636–1652. 10.1101/gad.1558107 17606642 PMC1899473

[B152] Braendle C. Félix M. A. (2008). Plasticity and errors of a robust developmental system in different environments. Developmental Cell 15, 714–724. 19000836 10.1016/j.devcel.2008.09.011

[B13] Braunschweig U. Gueroussov S. Plocik A. M. Graveley B. R. Blencowe B. J. (2013). Dynamic integration of splicing within gene regulatory pathways. Cell 152, 1252–1269. 10.1016/j.cell.2013.02.034 23498935 PMC3642998

[B14] Brayer K. J. Lynch V. J. Wagner G. P. (2011). Evolution of a derived protein-protein interaction between HoxA11 and Foxo1a in mammals caused by changes in intramolecular regulation. Proc. Natl. Acad. Sci. U.S.A. 108, E414–E420. 10.1073/pnas.1100990108 21788518 PMC3156161

[B15] Britten R. J. (1982). Genomic alterations in evolution, in Evolution and Development Dahlem Workshop Reports, ed Bonner J. T. (Berlin Heidelberg: Springer), 41–64.

[B16] Britten R. J. Davidson E. H. (1969). Gene regulation for higher cells: a theory. Science 165, 349–357. 10.1126/science.165.3891.349 5789433

[B17] Buljan M. Chalancon G. Dunker A. K. Bateman A. Balaji S. Fuxreiter M.. (2013). Alternative splicing of intrinsically disordered regions and rewiring of protein interactions. Curr. Opin. Struct. Biol. 23, 443–450. 10.1016/j.sbi.2013.03.006 23706950

[B18] Buljan M. Chalancon G. Eustermann S. Wagner G. P. Fuxreiter M. Bateman A.. (2012). Tissue-specific splicing of disordered segments that embed binding motifs rewires protein interaction networks. Mol. Cell 46, 871–883. 10.1016/j.molcel.2012.05.039 22749400 PMC3437557

[B19] Cadigan K. M. Nusse R. (1997). Wnt signaling: a common theme in animal development. Genes Dev. 11, 3286–3305. 9407023 10.1101/gad.11.24.3286

[B20] Campbell K. M. Terrell A. R. Laybourn P. J. Lumb K. J. (2000). Intrinsic structural disorder of the C-terminal activation domain from the bZIP transcription factor Fos. Biochemistry 39, 2708–2713. 10.1021/bi9923555 10704222

[B21] Caporael L. R. Griesemer J. R. Wimsatt W. C. (Eds.). (2014). Developing Scaffolds in Evolution, Culture, and Cognition. Cambridge, MA: MIT Press.

[B22] Capovilla M. Eldon E. D. Pirrotta V. (1992). The giant gene of *Drosophila* encodes a b-ZIP DNA-binding protein that regulates the expression of other segmentation gap genes. Development 114, 99–112. 1576969 10.1242/dev.114.1.99

[B23] Carroll S. B. Greniwer J. Weatherbee S. (2004). From DNA to Diversity: Molecular Genetics and the Evolution of Animal Design. Malden, MA: Blackwell Publishing.

[B24] Catania F. Lynch M. (2008). Where do introns come from? PLoS Biol. 6:e283. 10.1371/journal.pbio.0060283 19067485 PMC2586383

[B25] Chang C.-Y. Lin W.-D. Tu S.-L. (2014). Genome-wide analysis of heat-sensitive alternative splicing in *Physcomitrella patens*. Plant Physiol. 165, 826–840. 10.1104/pp.113.230540 24777346 PMC4044832

[B26] Chen C. D. Kobayashi R. Helfman D. M. (1999). Binding of hnRNP H to an exonic splicing silencer is involved in the regulation of alternative splicing of the rat?α-tropomyosin gene. Genes Dev. 13, 593–606. 10.1101/gad.13.5.593 10072387 PMC316507

[B27] Chen L. Bush S. J. Tovar-Corona J. M. Castillo-Morales A. Urrutia A. O. (2014a). Correcting for differential transcript coverage reveals a strong relationship between alternative splicing and organism complexity. Mol. Biol. Evol. 31, 1402–1413. 10.1093/molbev/msu083 24682283 PMC4032128

[B28] Chen L. Kostadima M. Martrens J. H. Canu G. Garcia S. P. Turro E.. (2014b). Transcriptional diversity during lineage commitment of human blood progenitors. Science 345:1251033. 10.1126/science.1251033 25258084 PMC4254742

[B29] Chow L. T. Gelinas R. E. Broker T. R. Roberts R. J. (1977). An amazing sequence arrangement at the 5′ ends of adenovirus 2 messenger RNA. Cell 12, 1–8. 10.1016/0092-8674(77)90180-5 902310

[B30] Colak R. Kim T. Michaut M. Sun M. Irimia M. Bellay J.. (2013). Distinct types of disorder in the human proteome: functional implications for alternative splicing. PLoS Comput. Biol. 9:e1003030. 10.1371/journal.pcbi.1003030 23633940 PMC3635989

[B31] Davidson E. H. (1982). Evolutionary change in genomic regulatory organization: speculations on the origins of novel biological structure, in Evolution and Development. Dahlem Workshop Reports, ed Bonner J. T. (Heidelberg: Springer Berlin), 65–84.

[B32] Davidson E. H. Erwin D. H. (2006). Gene regulatory networks and the evolution of animal body plans. Science 311, 796–800. 10.1126/science.1113832 16469913

[B33] De Masi F. Grove C. A. Vedenko A. Alibes A. Gisselbrecht S. S. Serrano L.. (2011). Using a structural and logics systems approach to infer bHLH-DNA binding specificity determinants. Nucleic Acids Res. 39, 4553–4563. 10.1093/nar/gkr070 21335608 PMC3113581

[B97] de Navas L. F. de Reed H. Akam M. Barrio R. Alonso C. R. Sánchez-Herrero E.. (2011). Integration of RNA processing and expression level control modulates the function of the *Drosophila* Hox gene Ultrabithorax during adult. Development 138, 107–116. 10.1242/dev.051409 21115609

[B34] Ducas V. C. Rhoades E. (2014). Investigation of intramolecular dynamics and conformations of α-, β-, and γ-Synuclein. PLoS ONE 9:e86983. 10.1371/journal.pone.0086983 24489820 PMC3904966

[B35] Dunker A. K. Bondos S. E. Huang F. Oldfield C. J. (2014). Intrinsically disordered proteins and multicellular organisms. Semin. Cell Devel. Biol. [Epub ahead of print]. 10.1016/j.semcdb.2014.09.025 25307499

[B36] Dunker A. K. Silman I. Uversky V. N. Sussman J. L. (2008). Function and structure of inherently disordered proteins. Curr. Opin. Struct. Biol. 18, 756–764. 10.1016/j.sbi.2008.10.002 18952168

[B37] Dunker A. K. Uversky V. N. (2010). Understanding protein non-folding. Biochim. Biophys. Acta 1804, 1231–1264. 10.1016/j.bbapap.2010.01.017 20117254 PMC2882790

[B38] Dyson H. J. Wright P. E. (2005). Intrinsically unstructured proteins and their functions. Nature Rev. Mol. Cell Biol. 6, 197–208. 10.1038/nrm1589 15738986

[B39] Ellis J. D. Barrios-Rodiles M. Colak R. Irimia M. Kim T. Calarco J. A.. (2012). Tissue-specific alternative splicing remodels protein-protein interaction networks. Mol. Cell. 46, 884–892. 10.1016/j.molcel.2012.05.037 22749401

[B40] Elowitz M. B. Levine A. J. Siggia E. D. Swain P. S. (2002). Stochastic gene expression in a single cell. Science 297, 1183–1186. 10.1126/science.1070919 12183631

[B41] Fairbrother W. G. Holste D. Burge C. B. Sharp P. A. (2004). Single nucleotide polymorphism–based validation of exonic splicing enhancers. PLoS Biol. 2:e268. 10.1371/journal.pbio.0020268 15340491 PMC514884

[B42] Forgacs G. Newman S. A. (2005). Biological Physics of the Developing Embryo. New York, NY: Cambridge University Press 10.1017/CBO9780511755576

[B43] Foster D. V. Foster J. G. Huang S. Kauffman S. A. (2009). A model of sequential branching in hierarchical cell fate determination. J. Theor. Biol. 260, 589–597. 10.1016/j.jtbi.2009.07.005 19615382

[B44] Fuxreiter M. Simon I. Bondos S. (2011). Dynamic protein–DNA recognition: beyond what can be seen. Trends Biochem. Sci. 36, 415–423. 10.1016/j.tibs.2011.04.006 21620710

[B45] Galant R. Carroll S. B. (2002). Evolution of a transcriptional repression domain in an insect Hox protein. Nature 415, 910–913. 10.1038/nature717 11859369

[B46] Galbraith M. D. Saxton J. Li L. Shelton S. J. Zhang H. Espinosa J. M. Shaw P. E.. (2013). ERK phosphorylation of MED14 in promoter complexes during mitogen-induced gene activation by Elk-1. Nucleic Acids Res. 41, 10241–10253. 10.1093/nar/gkt837 24049075 PMC3905876

[B47] Gao J. Thelen J. J. Dunker A. K. Xu D. (2010). Musite, a tool for global prediction of general and kinase-specific phosphorylation sites. Mol. Cell. Proteomics 9, 2586–2600. 10.1074/mcp.M110.001388 20702892 PMC3101956

[B48] Gao J. Xu D. (2012). Correlation between posttranslational modification and intrinsic disorder in protein. Pac. Symp. Biocomput. 2012, 94–103 10.1142/9789814366496_0010 22174266 PMC5120255

[B49] Gavis E. R. Hogness D. S. (1991). Phosphorylation, expression and function of the Ultrabithorax protein family in *Drosophila melanogaster*. Development 112, 1077–1093. 1682129 10.1242/dev.112.4.1077

[B50] Glass L. (1975). Classification of biological networks by their qualitative dynamics. J. Theor. Biol. 54, 85–107. 1202295 10.1016/s0022-5193(75)80056-7

[B51] Glass L. Kauffman S. A. (1973). The logical analysis of continuous, non-linear biochemical control networks. J. Theor. Biol. 39, 103–129. 4741704 10.1016/0022-5193(73)90208-7

[B52] Gomez D. Marathe R. Bierbaum V. Klumpp S. (2014). Modeling stochastic gene expression in growing cells. J. Theor. Biol. 348, 1–11. 10.1016/j.jtbi.2014.01.017 24480713

[B53] Gonzalez D. Hamidi N. Del Sol R. Benschop J. J. Nancy T. Li C.. (2014). Suppression of mediator is regulated by Cdk8-dependent Grr1 turnover of the Med3 coactivator. Proc. Natl. Acad. Sci. U.S.A. 111, 2500–2505. 10.1073/pnas.1307525111 24550274 PMC3932902

[B54] Grenier J. K. Carroll S. B. (2000). Functional evolution of the Ultrabithorax protein. Proc. Natl. Acad. Sci. U.S.A. 97, 704–709. 10.1073/pnas.97.2.704 10639143 PMC15394

[B55] Haag E. S. (2014). The same but different: worms reveal the pervasiveness of developmental system drift. PLoS Genet. 10:e1004150. 10.1371/journal.pgen.1004150 24516407 PMC3916242

[B56] Hasty J. Collins J. J. (2001). Protein interactions. Unspinning the web. Nature 411, 30–31. 10.1038/35075182 11333958

[B57] Haynes C. Iakoucheva L. M. (2006). Serine/arginine-rich splicing factors belong to a class of intrinsically disordered proteins. Nucleic Acids Res. 34, 305–312. 10.1093/nar/gkj424 16407336 PMC1326245

[B58] Hernández- Hernández V. Niklas K. J. Newman S. A. (2012). Dynamical patterning modules in plant development and evolution. Int. J. Del. Biol. 56, 661–674. 10.1387/ijdb.120027mb 23319343

[B59] Hsiao H.-C. Gonzalez K. L. Catanese D. J. Jr. Jordy K. E. Matthews K. S. Bondos S. E. (2014). The intrinsically disordered regions of the Drosophila melanogaster Hox protein Ultrabithorax select interaction proteins based on partner topology. PLoS ONE 9:e108217. 10.1371/journal.pone.0108217 25286318 PMC4186791

[B60] Hsu W.-L. Oldfield C. J. Xue B. Meng J. Huang F. Romero P.. (2013). Exploring the binding diversity of intrinsically disordered proteins involved in one-to-many binding. Protein Sci. 22, 258–273. 10.1002/pro.2207 23233352 PMC3595456

[B61] Iakoucheva L. M. Radivojac P. Brown C. J. O'Connor T. R. Sikes J. G. Obradovic Z.. (2004). The importance of intrinsic disorder for protein phosphorylation. Nucleic Acids Res. 32, 1037–1049. 10.1093/nar/gkh253 14960716 PMC373391

[B62] Ilsley G. R. Fisher J. Apweiler R. Depace A. H. Luscombe N. M. (2013). Cellular resolution models for even skipped regulation in the entire *Drosophila* embryo. eLife 2:e00522. 10.7554/eLife.00522 23930223 PMC3736529

[B63] Jacobs F. Schreiber S. (2006). Random perturbations of dynamical systems with absorbing states. SIAM J. Appl. Dyn. Syst. 5, 293–312 10.1137/050626417

[B64] Jaeger J. Monk N. (2014). Bioattractors: dynamical systems theory and the evolution of regulatory processes. J. Physiol. 592, 2267–2281. 10.1113/jphysiol.2014.272385 24882812 PMC4048087

[B65] Johnson F. B. Parker E. Krasnow M. A. (1995). Extradenticle protein is a selective cofactor for the *Drosophila* homeotics: role of the homeodomain and YPWM amino acid motif in the interaction. Proc. Natl. Acad. Sci. U.S.A. 92, 739–743. 10.1073/pnas.92.3.739 7846045 PMC42695

[B66] Johnson J. M. Castle J. Garrett-Engele P. Kan Z. Loerch P. M. Armour C. D.. (2003). Genome-wide survey of human alternative pre-mRNA splicing with exon junction microarrays. Science 302, 2141–2144. 10.1126/science.1090100 14684825

[B67] Kaganovich M. Snyder M. (2012). Phosphorylation of yeast transcription factors correlates with the evolution of novel sequence and function. J. Proteome Res. 11, 261–268. 10.1021/pr201065k 22141333 PMC4077355

[B68] Kaneko K. (2006). Life: an Introduction to Complex Systems Biology. New York, NY: Springer.

[B69] Kauffman S. (1969). Metabolic stability and epigenesis in randomly constructed genetic nets. J. Theor. Biol. 22, 437–467. 5803332 10.1016/0022-5193(69)90015-0

[B70] Kauffman S. (1974). The large scale structure and dynamics of gene control circuits: An ensemble approach. J. Theor. Biol. 44, 167–190. 4595774 10.1016/s0022-5193(74)80037-8

[B71] Ke S. Zhang X. H. Chasin L. A. (2008). Positive selection acting on splicing motifs reflects compensatory evolution. Genome Res. 18, 533–543. 10.1101/gr.070268.107 18204002 PMC2279241

[B72] Kim Y. Coppey M. Grossman R. Ajuria L. Jiménez G. Paroush Z. (2010). MAPK substrate competition integrates patterning signals in the *Drosophila* embryo. Curr. Biol. CB 20, 446–451 10.1016/j.cub.2010.01 PMC2846708 20171100

[B73] Kupiec J. (2009). The Origin of Individuals. New Jersey, NJ: World Scientific Publishing Company.

[B74] Labadorf A. Link A. Rogers M. F. Thomas J. Reddy A. S. Ben-Hur A.. (2010). Genome- wide analysis of alternative splicing in *Chlamydomonas reinhardtii*. BMC Genomics 11:114. 10.1186/1471-2164-11-114 20163725 PMC2830987

[B75] Lauffenburger D. A. (2000). Cell signaling pathways as control modules: complexity for simplicity? Proc. Natl. Acad. Sci. U.S.A. 97, 5031–5033. 10.1073/pnas.97.10.5031 10805765 PMC33983

[B76] Lavelle C. (2014). Pack, unpack, bend, twist, pull, push: the physical side of gene expression. Curr. Opin. Genet. Dev. 25, 74–84. 10.1016/j.gde.2014.01.001 24576847

[B77] Lee G. M. Pufall M. A. Meeker C. A. Kang H.-S. Graves B. J. McIntosh L. P. (2008). The affinity of Ets-1 for DNA is modulated by phosphorylation through transient interactions of an unstructured region. J. Mol. Biol. 382, 1014–1030. 10.1016/j.jmb.2008.07.064 18692067 PMC4808631

[B78] Leff S. E. Rosenfeld M. G. (1986). Complex transcriptional units: Diversity in gene expression by alternative RNA processing. Annu. Rev. Biochem. 55, 1091–1117. 3017190 10.1146/annurev.bi.55.070186.005303

[B79] Lim K. H. Ferraris L. Filloux M. E. Raphael B. J. Fairbrother W. G. (2011). Using positional distribution to identify splicing elements and predict pre-mRNA processing defects in human genes. Proc. Natl. Acad. Sci. U.S.A. 108, 11093–11098. 10.1073/pnas.1101135108 21685335 PMC3131313

[B80] Liu J. Perumal N. B. Oldfield C. J. Su E. W. Uversky V. N. Dunker A. K. (2006). Intrinsic disorder in transcription factors. Biochemistry 45, 6873–6888. 10.1021/bi0602718 16734424 PMC2538555

[B81] Liu Y. Matthews K. S. Bondos S. E. (2008). Multiple intrinsically disordered sequences alter DNA binding by the homeodomain of the *Drosophila* Hox protein Ultrabithorax. J. Biol. Chem. 283, 20874–20887. 10.1074/jbc.M800375200 18508761 PMC2475714

[B82] Liu Y. Matthews K. S. Bondos S. E. (2009). Internal regulatory interactions determine DNA binding specificity by a Hox transcription factor. J. Mol. Biol. 390, 760–774. 10.1016/j.jmb.2009.05.059 19481089 PMC2739810

[B83] Lopez A. J. Hogness D. S. (1991). Immunochemical dissection of the Ultrabithorax homeoprotein family in *Drosophila melanogaster*. Proc. Natl. Acad. Sci. U.S.A. 88, 9924–9928. 1719557 10.1073/pnas.88.22.9924 PMC52839

[B84] Lothrop A. P. Torres M. P. Fuchs S. M. (2013). Deciphering post-translational modification codes. FEBS Lett. 587, 1247–1257. 10.1016/j.febslet.2013.01.047 23402885 PMC3888991

[B85] Lucchetta E. M. Vincent M. E. Ismagilov R. F. (2008). A precise Bicoid gradient is nonessential during cycles 11-13 for precise patterning in the *Drosophila* blastoderm. PLoS ONE 3:e3651. 10.1371/journal.pone.0003651 18989373 PMC2578877

[B86] Ludwig M. Z. Manu Kittler R. White K. P. Kreitman M. (2011). Consequences of eukaryotic enhancer architecture for gene expression dynamics, development, and fitness. PLoS Genet. 7:e1002364. 10.1371/journal.pgen.1002364 22102826 PMC3213169

[B87] Lynch V. J. May G. Wagner G. P. (2011). Regulatory evolution through divergence of a phosphoswitch in the transcription factor CEBPB. Nature 480, 383–386. 10.1038/nature10595 22080951

[B88] Masel J. Trotter M. V. (2010). Robustness and evolvability. Trends Genet. 26, 406–14. 10.1016/j.tig.2010.06.002 20598394 PMC3198833

[B89] Matera A. G. Wang Z. (2014). A day in the life of the spliceosome. Nature Rev. Mol. Cell Biol. 15, 108–121. 10.1038/nrm3742 24452469 PMC4060434

[B150] Matlin A. J. Clark F. Smith C. W. J. (2005). Understanding alternative splicing: towards a cellular code. Nature Rev. Mol. Cell Biol. 6, 386–398. 10.1038/nrm1645 15956978

[B90] Milanovic M. Kracht M. Schmitz M. L. (2014). The cytokine-induced conformational switch of nuclear factor κ B p65 is mediated by p65 phosphorylation. Biochem. J. 457, 401–413. 10.1042/BJ20130780 24175631

[B91] Mileo E. Lorenzi M. Erales J. Lignon S. Puppo C. Le Breton N.. (2013). Dynamics of the intrinsically disordered protein CP12 in its association with GAPDH in the green alga *Chlamydomonas reinhardtii*: a fuzzy complex. Mol. Biosyst. 9, 2869–2876. 10.1039/c3mb70190e 24056937

[B92] Minde D. P. Radi M. Forneris F. Maurice M. M. Rüdiger S. G. D. (2013). Large extent of disorder in Adenomatous Polyposis Coli offers a strategy for Wnt signaling against point mutations. PLoS ONE 8:e77257. 10.1371/journal.pone.0077257 24130866 PMC3793970

[B93] Minezaki Y. Homma K. Kinjo A. R. Nishikawa K. (2006b). Human transcription factors contain a high fraction of intrinsically disordered regions essential for transcriptional regulation. J. Mol. Biol. 359, 1137–1149. 10.1016/j.jmb.2006.04.016 16697407

[B94] Minezaki Y. Homma K. Nishikawa K. (2006a). Genome-wide survey of transcription factors in prokaryotes reveals many bacteria-specific families not found in archaea. DNA Res. 12, 269–280. 10.1093/dnares/dsi016 16769689

[B95] Mohan A. Oldfield C. J. Radivojac P. Vacic V. Cortese M. S. Dunker A. K.. (2006). Analysis of molecular recognition features (MoRFs). J. Mol. Biol. 362, 1043–1059. 10.1016/j.jmb.2006.07.087 16935303

[B96] Mooney S. M. Qiu R. Kim J. J. Sacho E. J. Rajagopalan K. Johng D.. (2014). Cancer/testis antigen PAGE4, a regulator of c-Jun transactivation, is phosphorylated by homeodomain-interacting protein kinase 1, a component of the stress-response pathway. Biochemistry 53, 1670–1679. 10.1021/bi500013w 24559171 PMC4198062

[B98] Newman S. A. Bhat R. (2009). Dynamical patterning modules: a “pattern language” for development and evolution of multicellular form. Int. J. Dev. Biol. 53, 693–705. 10.1387/ijdb.072481sn 19378259

[B99] Niklas K. J. Cobb E. D. Dunker A. K. (2014). The number of cell types, information content, and the evolution of multicellularity. Acta Soc. Bot. Poloniae 83, 337 –347 10.5586/asbp.2014.034

[B100] Niklas K. J. Kutschera U. (2012). Plant development, auxin, and the subsystem incompleteness theorem. Front. Plant Evol. Develop. 3:37. 10.3389/fpls.2012.00037 22645582 PMC3355799

[B101] Nusse R. Varmus H. (2012). Three decades of Wnts: a personal perspective on how a scientific field developed. EMBO J. 31, 2670–2684. 10.1038/emboj.2012.146 22617420 PMC3380217

[B102] O'Connor M. B. Binari R. Perkins L. A. Bender W. (1988). Alternative RNA products from the Ultrabithorax domain of the bithorax complex. EMBO J. 7, 435–445. 2452731 10.1002/j.1460-2075.1988.tb02831.x PMC454339

[B103] Oldfield C. J. Cheng Y. Cortese M. S. Romero P. Uversky V. N. Dunker A. K. (2005). Coupled folding and binding with α-helix-forming molecular recognition elements. Biochemistry 44, 12454–12470. 10.1021/bi050736e 16156658

[B104] Oldfield C. J. Dunker A. K. (2014). Intrinsically disordered protein and intrinsically disordered protein regions. Annu. Rev. Biochem. 83, 553–584. 10.1146/annurev-biochem-072711-164947 24606139

[B105] Oldfield C. J. Meng J. Yang J. Y. Yang M. Q. Uversky V. N. Dunker A. K.. (2008). Flexible nets: disorder and induced fit in the associations of p53 and 14-3-3 with their partners. BMC Genomics 9:S1. 10.1186/1471-2164-9-S1-S1 18366598 PMC2386051

[B106] Ollo R. Maniatis T. (1987). *Drosophila* Kruppel gene product produced in a baculovirus expression system is a nuclear phosphoprotein that binds to DNA. Proc. Natl. Acad. Sci. U.S.A. 84, 5700–5704. 3112773 10.1073/pnas.84.16.5700 PMC298930

[B107] Pan Q. Shai O. Lee L. J. Frey B. J. Blencowe B. J. (2008). Deep surveying of alternative splicing complexity in the human transcriptome by high-throughput sequencing. Nat. Genet. 40, 1413–1415. 10.1038/ng.259 18978789

[B108] Pearson J. C. Lemons D. McGinnis W. (2005). Modulating Hox gene functions during animal body patterning. Nature Rev. Genet. 6, 893–904. 10.1038/nrg1726 16341070

[B109] Pejaver V. Hsu W. L. Xin F. Dunker A. K. Uversky V. N. Radivojac P. (2014). The structural and functional signatures of proteins that undergo multiple events of post-translational modification. Protein Sci. 23, 1077–1093. 10.1002/pro.2494 24888500 PMC4116656

[B111] Ptashne M. (2004). A Genetic Switch: Phage Lambda Revisited. New York, NY: CSHL Press.

[B112] Reed H. C. Hoare T. Thomsen S. Weaver T. A. White R. A. H. Akam M. (2010). Alternative splicing modulates Ubx protein function in *Drosophila melanogaster*. Genetics 84, 745–758 10.1534/genetics.109.112086 20038634 PMC2845342

[B113] Richard M. Yvert G. (2014). How does evolution tune biological noise? Front. Genet. 5:374. 10.3389/fgene.2014.00374 25389435 PMC4211553

[B114] Romero P. R. Zaidi S. Fang Y. Y. Uversky V. N. Radivojac P. Cortese M.. (2006). Alternative splicing in concert with protein intrinsic disorder enables increased functional diversity in multicellular organisms. Proc. Natl. Acad. Sci. U.S.A. 103, 8390–8395. 10.1073/pnas.0507916103 16717195 PMC1482503

[B115] Ronshaugen M. McGinnis N. McGinnis W. (2002). Hox protein mutation and macroevolution of the insect body plan. Nature 415, 914–917. 10.1038/nature716 11859370

[B116] Rosenfeld N. Young J. W. Alon U. Swain P. S. Elowitz M. B. (2005). Gene regulation at the single-cell level. Science 307, 1962–1965. 10.1126/science.1106914 15790856

[B117] Rosenfeld N. Young J. W. Alon U. Swain P. S. Elowitz M. B. (2007). Accurate prediction of gene feedback circuit behavior from component properties. Mol.Syst. Biol. 3, 143. 10.1038/msb4100185 18004276 PMC2132446

[B118] Savageau M. A. (1974). Comparison of classical and autogenous systems of regulation in inducible operons. Nature 252, 546–549. 10.1038/252546a0 4431516

[B119] Schad E. Tompa P. Hegyi H. (2011). The relationship between proteome size, structural disorder and organism complexity. Genome Biol. 12:R120. 10.1186/gb-2011-12-12-r120 22182830 PMC3334615

[B120] Schnell S. Maini P. K. Newman T. J. Newman S. A. (eds.). (2008). Multiscale Models of Developmental Systems. San Diego, CA: Academic Press/Elsevier.

[B121] Shmulevich I. Kauffman S. A. Aldana M. (2005). Eukaryotic cells are dynamically ordered or critical but not chaotic. Proc. Natl. Acad. Sci. U.S.A. 102, 13439–13444. 10.1073/pnas.0506771102 16155121 PMC1224670

[B122] Simons S. S. Kumar R. (2013). Variable steroid receptor responses: Intrinsically disordered AF1 is the key. Mol. Cell Endocrinol. 376, 81–84. 10.1016/j.mce.2013.06.007 23792173 PMC3781172

[B123] Singh G. P. Dash D. (2007). Intrinsic disorder in yeast transcriptional regulatory network. Proteins 68, 602–605. 10.1002/prot.21497 17510967

[B124] Spellman R. Llorian M. Smith C. W. J. (2007). Crossregulation and functional redundancy between the splicing regulator PTB and its paralogs nPTB and ROD1. Mol. Cell 27, 420–434. 10.1016/j.molcel.2007.06.016 17679092 PMC1940037

[B125] Sprecher S. G. Desplan C. (2008). Switch of rhodopsin expression in terminally differentiated *Drosophila* sensory neurons. Nature 454, 533–537. 10.1038/nature07062 18594514 PMC2750042

[B126] Srinivasan N. Bhagawati M. Ananthanarayanan B. Kumar S. (2014). Stimuli-sensitive intrinsically disordered protein brushes. Nat. Commun. 5, 5145. 10.1038/ncomms6145 25312006

[B127] Steiner C. F. (2012). Environmental noise, genetic diversity and the evolution of evolvability and robustness in model gene networks. PLoS ONE 7:e52204. 10.1371/journal.pone.0052204 23284934 PMC3527431

[B128] Strahl B. D. Allis C. D. (2000). The language of covalent histone modifications. Nature 403, 41–45. 10.1038/47412 10638745

[B129] Strogatz S. H. (2001). Nonlinear Dynamics and Chaos: With Applications To Physics, Biology, Chemistry, and Engineering. Cambridge: Westview Press.

[B130] Sun X. Jones W. T. Harvey D. Edwards P. J. B. Pascal S. M. Kirk C.. (2010). N-terminal domains of DELLA proteins are intrinsically unstructured in the absence of interaction with GID1/gibberellic acid receptors. J. Biol. Chem. 285, 11557–11571. 10.1074/jbc.M109.027011 20103592 PMC2857034

[B131] Sun X. Rikkerink E. H. A. Jones W. T. Uversky V. N. (2013). Multifarious roles of intrinsic disorder in proteins illustrate its broad impact on plant biology. Plant Cell 25, 38–55. 10.1105/tpc.112.106062 23362206 PMC3584547

[B132] Sun X. Xue B. Jones W. T. Rikkerink E. Dunker A. K. Uversky V. N. (2011). A functionally required unfoldome from the plant kingdom: intrinsically disordered N-terminal domains of GRAS proteins are involved in molecular recognition during plant development. Plant Mol. Biol. 77, 205–223. 10.1007/s11103-011-9803-z 21732203

[B133] Taghli-Lamallem O. Hsia C. Ronshaugen M. McGinnis W. (2008). Context-dependent regulation of Hox protein functions by CK2 phosphorylation sites. Dev. Genes Evol. 218, 321–332. 10.1007/s00427-008-0224-1 18504607 PMC2443945

[B134] Talavera D. Robertson D. L. Lovell S. C. (2013). Alternative splicing and protein interaction data sets. Nat. Biotechnol. 31, 292–293. 10.1038/nbt.2540 23563420

[B135] Tan X.-X. Bondos S. Li L. Matthews K. S. (2002). Transcription activation by ultrabithorax Ib protein requires a predicted alpha-helical region. Biochemistry 41, 2774–2785. 10.1021/bi011967y 11851425

[B136] True J. R. Haag E. S. (2001). Developmental system drift and flexibility in evolutionary trajectories. Evol. Dev. 3, 109–119. 10.1046/j.1525-142x.2001.003002109.x 11341673

[B137] Uversky V. N. (2002). Natively unfolded proteins: a point where biology waits for a physics. Protein Sci. Publ. Protein Soc. 11, 739–756 10.1110/ps.4210102 PMC2373528 11910019

[B138] Van Roey K. Dinkel H. Weatheritt R. J. Gibson T. J. Davey N. E. (2013). The switches.ELM resource: a compendium of conditional regulatory interaction interfaces. Sci Signal. 6, rs7. 10.1126/scisignal.2003345 23550212

[B139] Vaquerizas J. M. Kummerfeld S. K. Teichmann S. A. Luscombe N. M. (2009). A census of human transcription factors: function, expression and evolution. Nature Rev. Genet. 10, 252–263. 10.1038/nrg2538 19274049

[B140] Vuzman D. Levy Y. (2012). Intrinsically disordered regions as affinity tuners in protein-DNA interactions. Mol. Biosyst. 8, 47–57. 10.1039/c1mb05273j 21918774

[B141] Walsh C. M. Carroll S. B. (2007). Collaboration between Smads and a Hox protein in target gene repression. Development 134, 3585–3592. 10.1242/dev.009522 17855427

[B151] Wang Z. Rolish M. E. Yeo G. Tung V. Mawson M.. (2004). Systematic identification and analysis of exonic splicing silencers. Cell 119, 831–845. 15607979 10.1016/j.cell.2004.11.010

[B142] Ward J. J. Sodhi J. S. McGuffin L. J. Buxton B. F. Jones D. T. (2004). Prediction and functional analysis of native disorder in proteins from the three kingdoms of life. J. Mol. Biol. 337, 635–645. 10.1016/j.jmb.2004.02.002 15019783

[B143] Xie H. Vucetic S. Iakoucheva L. M. Oldfield C. J. Dunker A. K. Uversky V. N.. (2007). Functional anthology of intrinsic disorder. 1. Biological processes and functions of proteins with long disordered regions. J. Proteome Res. 6, 1882–1898. 10.1021/pr060392u 17391014 PMC2543138

[B144] Xiong H. Y. Alipanahi B. Lee L. J. Bretschneider H. Merico D. Yuen R. K.. (2015). The human splicing code reveals new insights into the genetic determinants of disease. Science 347:1254806. 10.1126/science.1254806 25525159 PMC4362528

[B145] Xue B. Dunker A. K. Uversky V. N. (2012a). Orderly order in protein intrinsic disorder distribution: disorder in 3500 proteomes from viruses and the three domains of life. J. Biomol. Struct. Dyn. 30, 137–149. 10.1080/07391102.2012.675145 22702725

[B146] Xue B. Dunker A. K. Uversky V. N. (2012b). The roles of disorder in orchestrating the Wnt pathway. J. Biomol. Struct. Dyn. 29, 843–861. 10.1080/073911012010525024 22292947

[B147] Yogesha S. D. Mayfield J. E. Zhang Y. (2014). Cross-talk of phosphorylation and prolyl isomerization of the C-terminal domain of RNA polymerase II. Molecules 19, 1481–1511. 10.3390/molecules19021481 24473209 PMC4350670

[B148] Zaitlen N. Kraft P. (2012). Heritability in the genome-wide association era. Hum.Genet. 131, 1655–1664. 10.1007/s00439-012-1199-6 22821350 PMC3432754

[B149] Zhao W. Li Y. (2011). Existence of random attractors for a p-Laplacian-type equation with additive noise. Abstr. Appl. Anal. 2011:e616451 10.1155/2011/616451

